# Six-pack holography for dynamic profiling of thick and extended objects by simultaneous three-wavelength phase unwrapping with doubled field of view

**DOI:** 10.1038/s41598-023-45237-6

**Published:** 2023-11-07

**Authors:** Simcha K. Mirsky, Natan T. Shaked

**Affiliations:** Department of Biomedical Engineering, Tel Aviv University, 69978 Tel Aviv, Israel

**Keywords:** Interference microscopy, Optical metrology, Biomedical engineering

## Abstract

Dynamic holographic profiling of thick samples is limited due to the reduced field of view (FOV) of off-axis holography. We present an improved six-pack holography system for the simultaneous acquisition of six complex wavefronts in a single camera exposure from two fields of view (FOVs) and three wavelengths, for quantitative phase unwrapping of thick and extended transparent objects. By dynamically generating three synthetic wavelength quantitative phase maps for each of the two FOVs, with the longest wavelength being 6207 nm, hierarchical phase unwrapping can be used to reduce noise while maintaining the improvements in the 2*π* phase ambiguity due to the longer synthetic wavelength. The system was tested on a 7 μm tall PDMS microchannel and is shown to produce quantitative phase maps with 96% accuracy, while the hierarchical unwrapping reduces noise by 93%. A monolayer of live onion epidermal tissue was also successfully scanned, demonstrating the potential of the system to dynamically decrease scanning time of optically thick and extended samples.

## Introduction

Histologically stained tissue biopsies are scanned routinely for medical diagnosis using brightfield microscopy. Holography, a quantitative label-free microscopy technique, does not require tissue staining and can provide rich data suitable for automated deep learning analysis^1–6^ and grants access to new metrics such as local dry mass^7–9^. This is done by acquiring the quantitative phase profile of the sample which corresponds to the refractive index of the sample at all points in the image. Limitations of holography include the need to acquire multiple images when using sequential on-axis holography^10–12^ or the need to reduce the field of view (FOV) in off-axis holography^13^. The latter is suitable for dynamic samples and fast scanning since only a single camera exposure is needed to reconstruct the quantitative phase profile of the sample. However, the FOV must be reduced by a factor of four across each axis (1/16 of the image area) compared to brightfield microscopy of the same resolution in order to acquire the off-axis fringes of the hologram while avoiding overlap of terms in the spatial frequency domain^13^. Furthermore, the quantitative phase images obtained from holography are wrapped within a range of [‒ *π*,*π*) radians, such that for optically thick samples the resulting phase images may often possess inaccurate values due to 2*π* phase ambiguities, even after applying digital unwrapping algorithms^14–16^. The latter issue can be solved using two-wavelength phase unwrapping^17–21^, in which two holograms of different wavelengths are acquired in order to generate a single quantitative phase map corresponding to a synthetic wavelength that is much longer than any of the illumination wavelengths, and thus avoids phase unwrapping problems. This synthetic wavelength phase map will also have correspondingly increased noise due to the differential process of creating the synthetic wavelength, which may be improved by using hierarchical phase unwrapping to compare the synthetic wavelength map to the original illumination wavelength phase maps^22^. However, two-wavelength phase unwrapping may still be prone to significant increases in noise and unwrapping errors when the magnitude of the noise in the synthetic wavelength phase map is greater than the phase value corresponding to half the illumination wavelength of the phase map to which the synthetic wavelength map is being compared. This issue may be solved via three-wavelength phase unwrapping^23,24^, in which the third wavelength is used to create two additional synthetic wavelengths to bridge the gap.

Simultaneous interferometric acquisition of the sample under two or three wavelengths^18,23–29^ via holographic multiplexing is suitable for dynamic samples that might otherwise change or move between sequential acquisitions of the different images for each illumination wavelength. Off-axis holographic multiplexing can be used to acquire six interference channels simultaneously via six-pack holography (6PH), a technique first described by Moran et al. in 2017^30^. In that initial work, it was demonstrated that 6PH was possible for holographic compression and that 6PH may be applied to optical acquisition of holograms using a Mach–Zehnder interferometer setup, however 6PH with an imaged sample was not experimentally demonstrated as there would be crosstalk between the 12 different beams, and instead only one empty sample beam and 6 reference beams were used. In our later works, using a low-coherence light source and echelons, crosstalk between the 6 or more different sample beams and 6 reference beams was prevented by coherence gating, enabling the experimental validation of 6PH and its application to synthetic aperture super-resolution^31^, out-of-focus light rejection^32^, and tomographic interferometry for 3D refractive index reconstruction^33^ using different Mach–Zehnder interferometer setups. Recently, we demonstrated the first external module for 6PH and its application for dynamic volumetry across two fields of view (FOVs)^29^.

In the present work, we utilize 6PH for the simultaneous off-axis holography of two FOVs simultaneously with three different wavelength channels per FOV for the application of hierarchical phase unwrapping. For this, we designed an improved external 6PH module that acquires two FOVs simultaneously and provides the ability to optically profile extended samples, or double scanning speeds. By simultaneous acquisition of three wavelengths per FOV, we generate synthetic wavelengths, enabling extraction of accurate phase values for optically thicker samples by hierarchical phase unwrapping.

## Methods

### Six-pack holography

**Figure 1:**
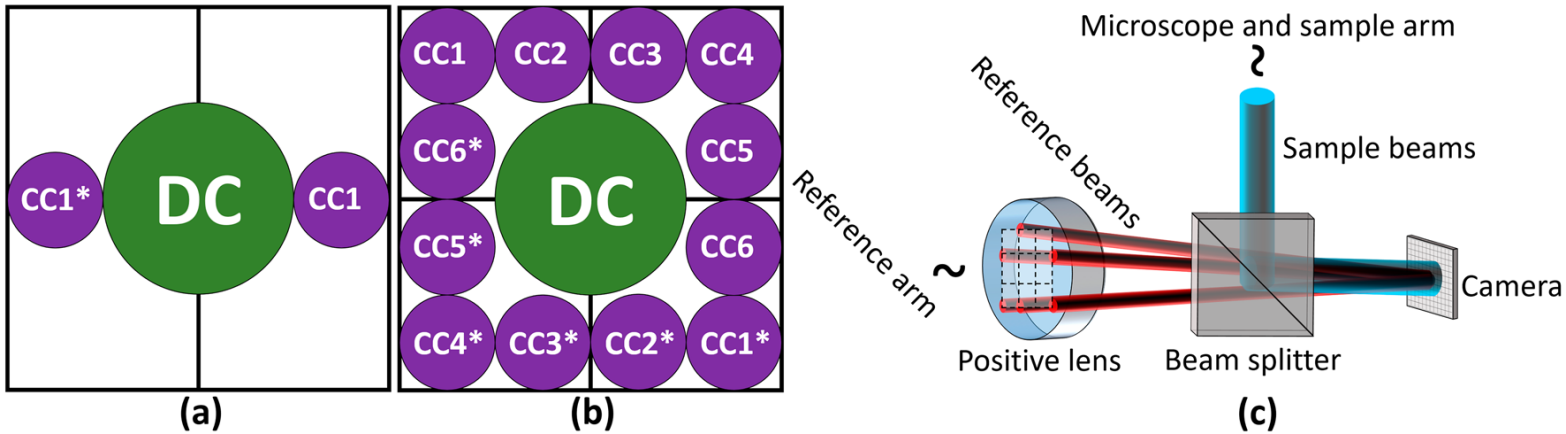
Comparison of the spatial frequency domain of 6PH to standard off-axis holography. (**a**) Standard off-axis holography, with a single CC term and corresponding complex conjugate term, designated as CC1 and CC1*. (**b**) Six-pack hologram, with six CC terms and matching complex conjugate terms, thereby containing six times more data per camera exposure in comparison to (**a**). (**c)** Simplified diagram of a 6PH system. CC, cross-correlation term; DC, autocorrelation term.

Holographic multiplexing enables the simultaneous acquisition of more than a single complex wavefront in a single camera exposure^34–36^. A standard off-axis hologram possesses parallel linear interference fringes, which act as a carrier frequency for the complex wavefront data of the sample, represented by the corresponding cross-correlation (CC) terms in the spatial frequency domain, which prevents overlap with the central autocorrelation (DC) term and enables reconstruction of the sample complex wavefront from a single image^37^, as shown in Fig. 1a. Angular or spatial frequency multiplexing by projecting multiple sample and reference beam pairs onto the camera with different transverse beam orientations enables simultaneous generation of interference fringes of different orientations, all sharing the dynamic range of the camera. 6PH applies this technique to multiplex six different complex wavefronts, with the off-axis angles carefully selected to prevent overlap between the resulting six CC-term pairs and the DC terms, enabling reconstruction of the six complex wavefronts from a single camera exposure or video frame, as shown in Fig. 1b. In a typical 6PH setup, six reference beams, generated using a diffractive beam splitter, arrive from the reference arm in parallel and are made to be collimated by a positive lens, while converging onto the camera at the six different off-axis angles required, as shown in Fig. 1c. At the positive lens, the six reference beams are arranged at selected vertices of a 3 × 3 grid of squares, such that no reference beam has another reference beam on the opposite side along the line that extends from the reference beam location on the grid through the optical axis in the center. The lack of opposing beams is necessary as the CC terms would otherwise overlap, and this square arrangement results in the square arrangement of the CC terms seen in Fig. 1b. The six sample beams then arrive from the sample arm and are combined with the reference beams by a beam splitter, creating the six-pack hologram on the camera. As 6PH requires a total of 12 different beams, 6 pairs of sample and reference beams, crosstalk interference between non-matching beam pairs would typically occur, which would lead to additional unwanted and overlapping terms that would prevent accurate reconstruction. In this work, crosstalk is prevented by two techniques. First, crosstalk will not occur between light of different wavelengths, thus the three wavelength channels per FOV will not experience crosstalk. Second, all three sample beams per FOV, and their corresponding three reference beams, are orthogonally polarized relative to the sample and reference beams of the other FOV, thereby preventing interference between these two sets of beams. Once the six-pack hologram has been acquired, the complex wavefront images of the three different wavelength holograms for each of the two FOVs are digitally reconstructed by applying a Fourier transform (FT), cropping out the six corresponding CC terms, and performing an inverse FT (IFT). The quantitative phase maps extracted from the complex wavefront images from the three wavelength channels for each FOV were then used to generate synthetic wavelength phase maps.

### Synthetic-wavelength phase unwrapping

In standard digital unwrapping algorithms^38^, the algorithm only attempts to detect the locations of 2*π* phase discontinuities in the wrapped phase map and to correct them by adding 2*π* radians to the corresponding region, and thus cannot correct for discontinuities greater than 2*π* radians, as any phase value could represent that same value plus an integer multiple of 2*π* radians, the phenomenon known as 2*π* phase ambiguity. Synthetic-wavelength phase unwrapping mitigates the problem of 2*π* phase ambiguity by acquiring the sample under two wavelengths, and synthetically increasing the wavelength of the phase by subtracting the two phase profiles, so that 2*π* ambiguities are avoided; however, the phase noise increases correspondingly. Hierarchical phase unwrapping resolves the problem of increased phase noise in synthetic-wavelength phase unwrapping by comparing the optical path delay (OPD) map of the longest-wavelength unwrapped phase map, which is in this case the noisy synthetic-wavelength map, to the much less noisy shorter-wavelength non-synthetic OPD maps, in order to generate a corresponding denoised phase map, as described in greater detail in the following section.

In synthetic-wavelength phase unwrapping, by subtracting the quantitative phase maps of different wavelengths we can produce a new quantitative phase map corresponding to the wavelength of the beat frequency of the original two wavelengths^17–21^. The difference of two phase maps, ${\varphi }_{D}={\varphi }_{1}-{\varphi }_{2}$, corresponds to a synthetic wavelength phase map possessing a synthetic wavelength equal to ${\Lambda }_{D}={\lambda }_{1}{\lambda }_{2}/\left({\lambda }_{2}{-\lambda }_{1}\right)$, where *λ*_1_ is the wavelength of phase map *φ*_1_ and is shorter than *λ*_2_, the wavelength of phase map *φ*_2_. This results in a synthetic wavelength longer than either of the original two wavelengths. The sum of two phase maps, ${\varphi }_{S}={\varphi }_{1}+{\varphi }_{2}$, corresponds to a synthetic wavelength phase map possessing a synthetic wavelength equal to ${\Lambda }_{s}={\lambda }_{1}{\lambda }_{2}/\left({\lambda }_{1}{+\lambda }_{2}\right)$, resulting in a synthetic wavelength shorter than either of the original two wavelengths. A longer synthetic wavelength will have wrapped phase in the range of 2*π* radians according to its wavelength, such that the maximum optical path delay of the sample that may be acquired accurately without requiring phase unwrapping is increased. The OPD is defined as:

1 $$OPD=\left({n}_{s}-{n}_{m}\right)h= {\varphi }_{n}\frac{{\lambda }_{n}}{2\pi },$$

where *n*_s_ is the integral refractive index of the sample along the optical axis, *n*_m_ is the refractive index of the surrounding medium, and *h* is the height or thickness of the sample. Using the longer synthetic wavelength phase map, the OPD value for a phase of 2*π* radians correspondingly increases, such that for lower OPDs one may treat the wrapped phase map, or the corresponding OPD map, as being unwrapped. However, it is important to note that the noise of the synthetic-wavelength phase map, and resulting OPD map, are correspondingly increased by the same factor that the wavelength is increased (or decreased as the wavelength is decreased, in the case of synthetic wavelengths acquired by summation). To reduce the noise present in the longest synthetic wavelength phase map, hierarchical phase unwrapping can be used^22^.

### Hierarchical phase unwrapping

**Figure 2:**
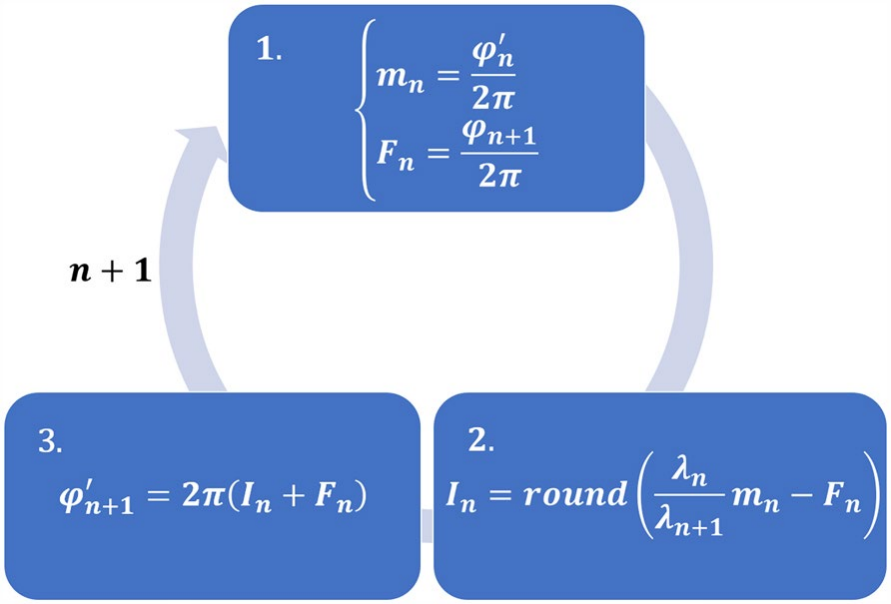
Hierarchical phase unwrapping algorithm. ${\varphi }_{n}^{\mathrm{^{\prime}}}$, current longest-wavelength unwrapped phase map; ${m}_{n}$, phase order of ${\varphi }_{n}^{\mathrm{^{\prime}}}$; ${\varphi }_{n+1}$, next-longest wrapped phase map; ${F}_{n}$, fractional part of ${\varphi }_{n+1}$; ${\lambda }_{n}$, wavelength of ${\varphi }_{n}^{\mathrm{^{\prime}}}$ and ${\varphi }_{n}$; ${\lambda }_{n+1}$, wavelength of ${\varphi }_{n+1}^{\mathrm{^{\prime}}}$ and ${\varphi }_{n+1}$; ${I}_{n}$, the estimated integer part.

Hierarchical phase unwrapping essentially compares the longest unwrapped OPD map to the next-longest wrapped OPD map and adds or subtracts integer multiples of the wavelength of the shorter and wrapped OPD map to the OPD value of each pixel of that same map, thereby unwrapping the shorter-wavelength OPD map and the corresponding phase map. The algorithm is outlined in Fig. 2, where in Step 1 the phase order, ${m}_{n}$, of the current longest-wavelength unwrapped phase map, ${\varphi }_{n}{\prime}$, is calculated, as well as the fractional part, ${F}_{n}$, of the next-longest wrapped phase map, ${\varphi }_{n+1}$. In Step 2, the difference between the two OPDs of ${m}_{n}$ and ${F}_{n}$ is calculated and divided by the wavelength of ${F}_{n}$, ${\lambda }_{n+1}$, and rounded to estimate the integer part, ${I}_{n}$, of this difference. Finally, in Step 3, ${I}_{n}$ is added to ${F}_{n}$ and multiplied by 2*π* to produce the unwrapped phase map of ${\varphi }_{n+1}$, ${\varphi }_{n+1}{\prime}$, and the process repeats until all phase maps have been unwrapped. By repeatedly comparing the longer-wavelength OPD map to next-longest OPD map and rounding to get the integer number of wavelengths for unwrapping, the noise in the resulting maps decreases step by step to correspond to the noise of the shortest-wavelength wrapped map, yet the OPD values remain approximately the same as the longest-wavelength OPD map, resulting in noise comparable to an OPD map extracted from a standard off-axis hologram, but without the wrapping problems, at least for sample OPD jumps that are less than the longest synthetic wavelength. This denoising effect of hierarchical phase unwrapping is limited by the noise level of the phase maps and the wavelengths used. If the noise of the unwrapped OPD map is greater than half the wavelength of the following wrapped map, the noise will not be properly rounded out, resulting in the erroneous addition or subtraction of a wavelength at that point. To prevent this, we acquired three different wavelengths images, thereby enabling the generation of three different synthetic wavelength phase maps by subtraction, such that the difference between the lengths of each successive wavelength is smaller, a technique previously demonstrated by Mann et al.^23^ and Turko et al.^24^.

### Optical system for parallel wavefront acquisition of three wavelength channels and two fields of view

**Figure 3:**
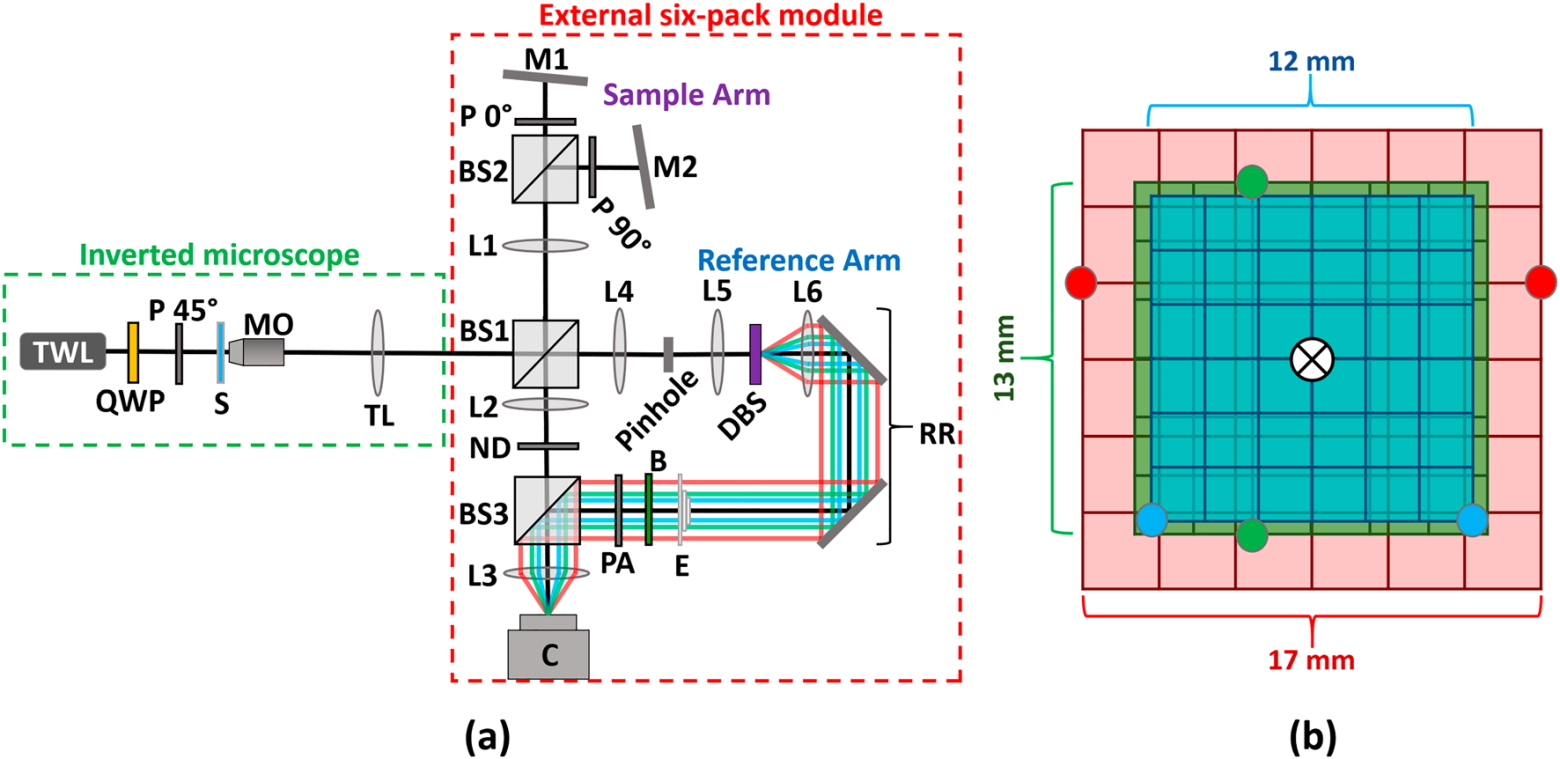
Six-pack holographic system. (**a**) System diagram. *TWL* three-wavelength light source; *QWP* quarter waveplate; P 45°, P 0°, P 90°: linear polarizers with the respective orientations; *S* sample; *MO* microscope objective lens; *TL* tube lens; BS1‒3: beam splitters; L1‒6: lenses; M1‒2: mirrors; *ND* neutral density filter; *DBS* diffractive beam splitter; *RR* retroreflector mirror; *E* echelon; *B* beam-stop; *PA* polarizing array. (**b**) Positions of the reference beams relative to the optical axis prior to L3. Red circles: 692 nm wavelength position; Green circles: 532 nm wavelength position; Blue circles: 490 nm wavelength position. ⦻ symbol indicates the optical axis.

The optical system for parallel wavefront acquisition of three wavelength channels and two FOVs is shown in Fig. 3a. The sample S is illuminated by a beam of linearly polarized partially-coherent light of three wavelengths, 692, 532, and 490 nm, produced by three-wavelength light source TWL (NKT SuperK EXTREME and three wavelengths selected by SuperK SELECT, with a maximum coherence length of 25.4 μm). A partially-coherent light source is used in order to reduce speckle noise. Prior to the sample, the light is linearly polarized while maintaining maximum possible intensity by passing through quarter waveplate QWL and linear polarizer P 45°, such that the angle of polarization is approximately ± 45° relative to the other polarizers in the system. The polarizer P 45° is an improvement upon our previous setup^29^, enabling equal assignment of intensity to the two orthogonally polarized sets of beams described later, thereby optimizing interference contrast and decreasing phase noise. The light passes through the sample and enters an inverted microscope comprised of microscope objective lens MO (Motic Plan Apo ELWD 20 × /0.42) and achromatic tube lens TL (focal length 200 mm). The resulting sample beam then enters the external 6PH module and is split into two beams by 50:50 beam splitter BS1. In the sample arm, the beam passes through achromatic lens L1 (focal length 150 mm) and is split again by 50:50 beam splitter BS2. The transmitted beam passes through linear polarizer P 0° and is reflected back through P 0° and BS2 by mirror M1, while the reflected beam passes through linear polarizer P 90° and is reflected back through P 90° and BS2 by mirror M2. Mirrors M1 and M2 are slightly tilted horizontally in opposite directions, causing the FOVs of the corresponding two sample beams to shift horizontally on the camera at the end, thereby overlaying two different FOVs in the camera plane. Polarizers P 0° and P 90° are at orthogonal polarization angles relative to each other, and at approximately 45° relative to P 45°, such that the two sample beams will not interfere with each other and will be at nearly identical intensities. Once the two sample beams have been recombined by BS2, they pass back through L1 and BS1, and then pass through achromatic lens L2 (focal length 75 mm), neutral density filter ND (optical density 0.8 or 1.1), 50:50 beam splitter BS3, and achromatic lens L3 (focal length 150 mm) to produce the three-wavelength images of the two different FOVs on the camera (FLIR GS3-U3-23S6M, 12-bit monochromatic CMOS, square pixels of 5.86 µm width and height), at a total magnification of 40× and with a maximal diffraction limited spot size of 2.01 µm. The filter ND is necessary to match the intensities of the sample and reference beams. A higher optical density was used for tissue samples, as the increased scattering caused decreased reference beam intensity relative to the sample beam intensity.

In the reference arm, the beam passes through achromatic lens L4 (focal length 75 mm), which is positioned at a distance of one focal length away from the following 30 µm pinhole, such that the pinhole is in the conjugate Fourier plane of the image from the microscope and thus acts as a low-pass spatial frequency filter, removing the sample information and creating a clean three-wavelength reference beam. The reference beam is then collimated by achromatic lens L5 (focal length 100 mm) and illuminates diffractive beam splitter DBS (DigitalOptics Corporation), generating three different 11 × 7 patterns of collimated beams (one pattern for each wavelength) diverging from the optical axis. These beams are then made parallel to the optical axis by achromatic lens L6 (focal length 200 mm), which is placed one focal length away from DBS, are reflected by the two mirrors in retroreflector RR, and pass through echelon E, such that the two desired beams of the 692 nm wavelength each pass through approximately 1.00 mm of glass, the two desired beams of the 532 nm wavelength each pass through approximately 3.30 mm of glass, and the two beams of the 490 nm wavelength each pass through approximately 4.15 mm of glass. This echelon is necessary to precisely match the optical path delays of the beams in order to obtain interference from all beams simultaneously on the camera plane, and has been improved in comparison to the previous work^29^ by increasing the thickness by approximately 3 mm in order to optimize the interference contrast and decrease phase noise. Next, all but the six desired reference beams illustrated in Fig. 3b are blocked by beam-stop B, and the beams pass through polarizing array PA, which polarizes half the beams (one of each wavelength) to match the polarization of P 0°, and half the beams to match the polarization of P 90°, thereby preventing unwanted interference between reference beams. The six reference beams are then combined with the sample beams as they pass through BS3 and L3 to produce six-pack off-axis interference on the camera. All lenses in the system are arranged in a 4f configuration, with the exception of L5 and L6, which have a distance of approximately 255 mm between them, in order to match the optical path delay of the sample and reference beam paths. The precise matching of the optical paths of the sample and reference beams is done using RR, which is mounted on a linear translation stage, allowing for precise control of the optical path delay of the reference arm.

### Phase retrieval & unwrapping algorithm

**Figure 4:**
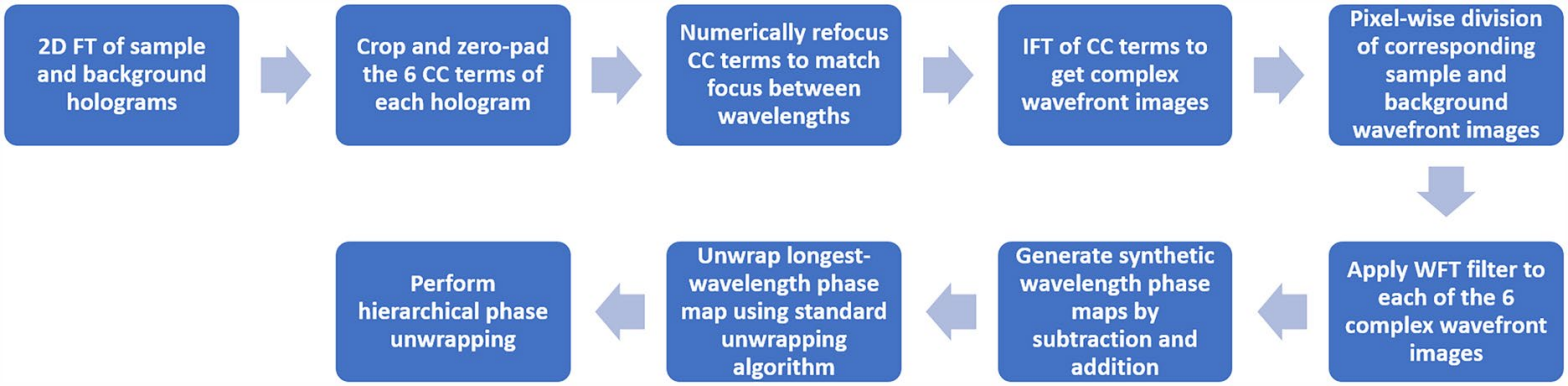
Phase retrieval algorithm.

The phase retrieval and unwrapping algorithm is outlined in Fig. 4. In order to extract the complex wavefront images from the acquired six-pack holograms, a 2D FT was applied to each hologram, and the six CC terms were cropped. Next, the CC terms were numerically refocused using Fresnel propagation^39^ in the Fourier domain, in order to correct for small changes in focus between wavelengths. The maximum refocus distance used was 11 μm. Following this, an IFT was applied to each CC term to reconstruct the six complex wavefront images. Each of the six complex wavefront images were then pixelwise divided by corresponding complex wavefront images from a background reference hologram containing no sample, only the surrounding medium. This process removes any constant phase curvatures from the complex wavefront image by subtracting the background image phase from the sample image phase. Lastly, a windowed FT (WFT) filter^40,41^ was applied to each of the six images to reduce high-frequency noise.

Once the complex wavefront images had been extracted and prepared, synthetic wavelength phase maps were generated by subtraction and by addition. For subtraction, the complex wavefront images of the three different wavelengths for each FOV were pixel-wise divided in order to take the difference of the phases in the complex exponents, whereas for addition the complex wavefront images of the three different wavelengths for each FOV were pixel-wise multiplied in order to sum the phases in the complex exponents. The synthetic wavelength phase was then extracted for each pixel in the image by taking the arctangent of the imaginary component divided by the real component. The synthetic wavelengths achieved by subtraction were thus: 6207 nm, by subtracting the phase of the 532 nm wavelength (green) image from the phase of the 490 nm wavelength (blue) image, 2301 nm by subtracting the phase of the 692 nm wavelength (red) image from the phase of the green image, and 1679 nm by subtracting the phase of the red image from the phase of the blue image. The synthetic wavelengths achieved by addition were: 300 nm, by adding the phase of the red image to the phase of the green image, 287 nm by adding the phase of the red image to the phase of the blue image, and 255 nm by adding the phase of the green image to the phase of the blue image. In total, this results in nine wrapped phase maps for each FOV, with wavelengths of 6207, 2301, 1679, 692, 532, 490, 300, 287, and 255 nm, respectively.

Following the generation of the nine wrapped phase maps, the longest-wavelength phase map (6207 nm) was unwrapped using a standard unwrapping algorithm by Miguel et al.^38^, and hierarchical phase unwrapping was performed as described in the above Synthetic-Wavelength Phase Unwrapping subsection.

## Results

**Figure 5:**
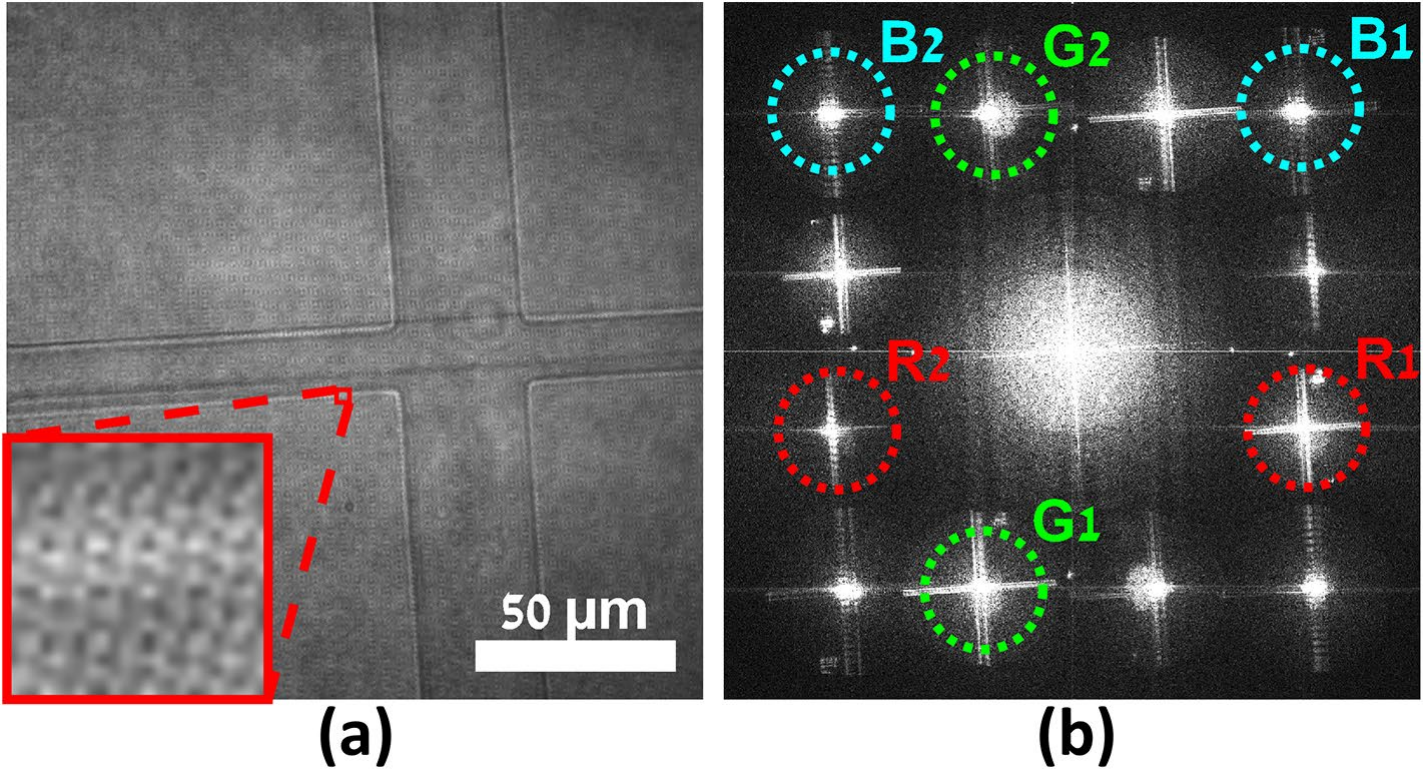
Six-pack hologram and corresponding spatial frequency power spectrum. (**a**) Six-pack hologram of microchannel. Inlay is magnified 20× to show fringes. (**b**) Spatial frequency power spectrum of (**a**) with CC term crop windows. R1, G1, B1: crop windows of 692, 532, and 490 nm wavelengths respectively, for left FOV. R2, G2, B2: crop windows of 692, 532, and 490 nm wavelengths respectively, for right FOV.

**Figure 6:**
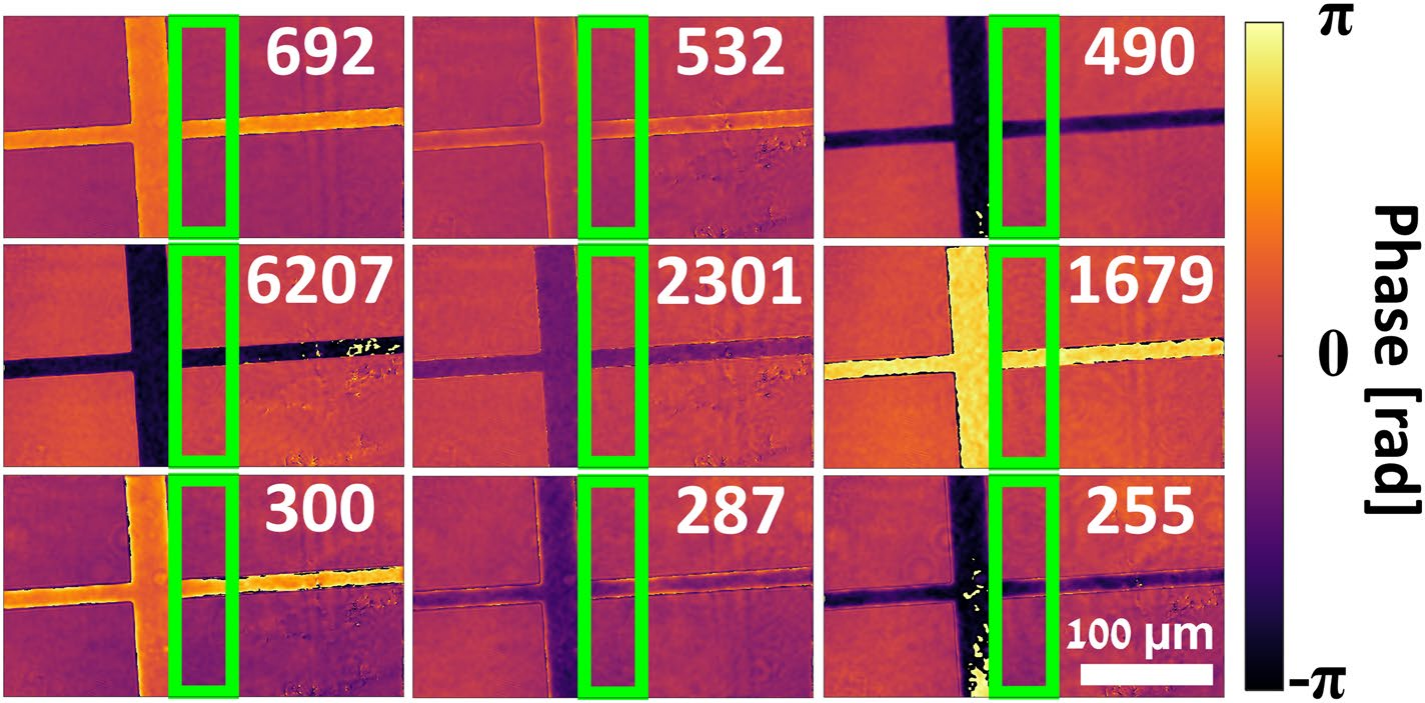
Wrapped phase maps of combined FOVs of microfluidic channel. Numbers in each image are the corresponding wavelength in nm. Top row: illumination wavelength phase maps, middle row: synthetic wavelength phase maps by subtraction, bottom row: synthetic wavelength phase maps by addition. Green rectangle indicates region of overlap between FOVs. Scale bar at bottom right applies to all phase maps.

The system was tested on a polydimethylsiloxane (PDMS) microchannel with a height of 7 μm containing only air. As the minimum refractive index of PDMS for the three illumination wavelengths is approximately 1.39^42^, the expected OPD of the air-filled microchannel relative to the surrounding medium of PDMS was (1.00–1.39) × 7 μm = ‒2730 nm, as per Eq. (1). The absolute value of this OPD is less than the synthetic wavelength of 6207 nm, yet greater than the synthetic wavelength of 2301 nm, making this a suitable target to test the hierarchical unwrapping capabilities of our system. The acquired six-pack hologram is shown in Fig. 5a, where it can be seen that two FOVs are overlaid; one FOV of the cross-shaped channel intersection, and the other of the continuation of the channel to the right, with three wavelength channels multiplexed per each FOV. The 2D FT of this hologram is shown in Fig. 5b, with the crop windows for each of the six CCs illustrated. A constant crop window matching the diameter of the 692 nm wavelength CC terms was used in order to avoid relative change in resolution between the wavelength channels, which otherwise could have caused errors during the hierarchical unwrapping process. The corresponding wrapped phase maps for the combined FOVs are shown in the top row of Fig. 6, while the synthetic-wavelength phase maps obtained by subtraction of the original phase maps are shown in the middle row of Fig. 6, and the synthetic-wavelength phase maps obtain by addition of the original phase maps are shown in the bottom row of Fig. 6. All phase maps are displayed using the perceptually uniform inferno colormap^43, 44^.

**Figure 7:**
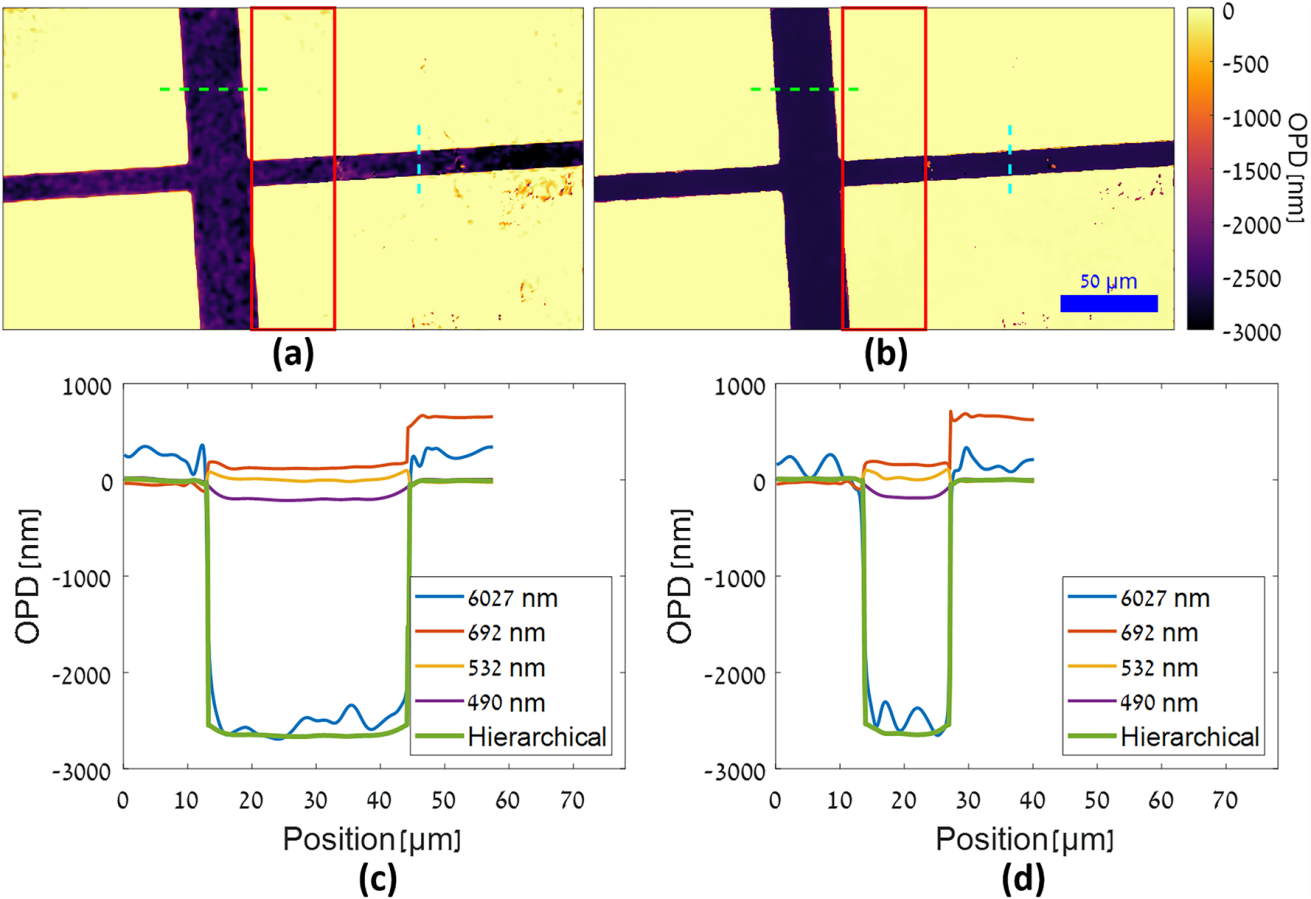
Unwrapped OPD maps of an air-filled PDMS microfluidic channel. (**a**) 6207 nm synthetic-wavelength OPD map, unwrapped by a standard unwrapper^38^. (**b**) Hierarchically-unwrapped OPD map. Scale and color bars apply to both OPD maps. Red rectangles indicate region of overlap between FOVs. (**c**) OPD value profile curves along dashed horizontal green lines in (**a**) and (**b**). (**d**) OPD value profile curves along dashed vertical cyan lines in (**a**) and (**b**).

The unwrapped OPD maps are shown in Fig. 7, with Fig. 7a showing the OPD map of the 6207 nm synthetic-wavelength channel, unwrapped by the standard digital unwrapper^38^, and Fig. 7b showing the final OPD map after hierarchical phase unwrapping starting from Fig. 7a. It can be seen in Fig. 7c and d that the hierarchically unwrapped OPD map is the most accurate and least noisy of the OPD maps, as the mean value inside the channel is approximately ‒2608 nm, which is 96% of the expected value, and the profile curve is smooth and level. The 6207 nm synthetic-wavelength standard-unwrapped OPD map shows significant noise in the profile curve, while the standard-unwrapped illumination-wavelength OPD maps show smoother profile curves but inaccurate OPD values for the channel. When using only the standard-unwrapped illumination-wavelength OPD maps, the closest OPD to the expected value of ‒2730 nm is seen in the 490 nm wavelength map, and its value is ‒179 nm, which is only 7% of the expected value. This result is to be expected, as due to the sharp change in phase the standard unwrapper misses the multiple wavelengths worth of phase due to 2*π* phase ambiguities. Furthermore, it can be seen that for the wavelength of 692 nm the OPD values outside the channel, corresponding to the bottom right region of PDMS, are actually greater than the values on the opposite side of the channel. This is due to the standard unwrapper erroneously interpreting the discontinuity at the channel edge as wrapped phase requiring the addition of 2*π*, an error which is avoided when using the hierarchical phase unwrapping algorithm.

Lastly, to quantify the relative improvement in noise for the hierarchically-unwrapped OPD map versus the standard-unwrapped OPD map, we took the standard deviations (std) of the same empty 200 × 200-pixel region in the OPD maps shown in Fig. 7a and b and found that the std value for the standard-unwrapped 6207 nm synthetic-wavelength OPD map was 55 nm, while the std value for the hierarchically-unwrapped OPD map was 4 nm, indicating a 93% reduction in OPD noise for the hierarchically-unwrapped version.

**Figure 8:**
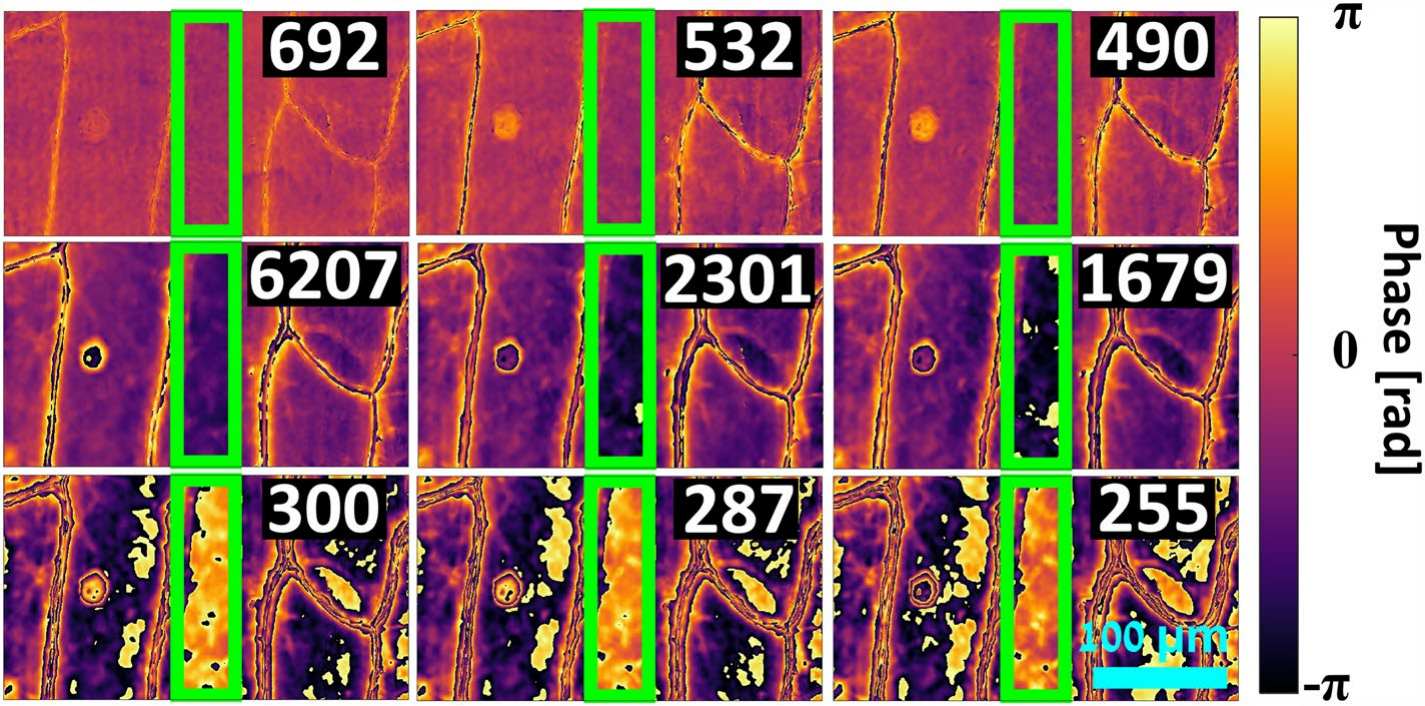
Wrapped phase maps of the combined FOVs of an onion epidermal tissue. Numbers in each image are the corresponding wavelength in nm. Top row: illumination wavelength phase maps, middle row: synthetic-wavelength phase maps by subtraction, bottom row: synthetic-wavelength phase maps by addition. Green rectangle indicates region of overlap between FOVs. Scale bar at bottom right applies to all phase maps.

**Figure 9:**
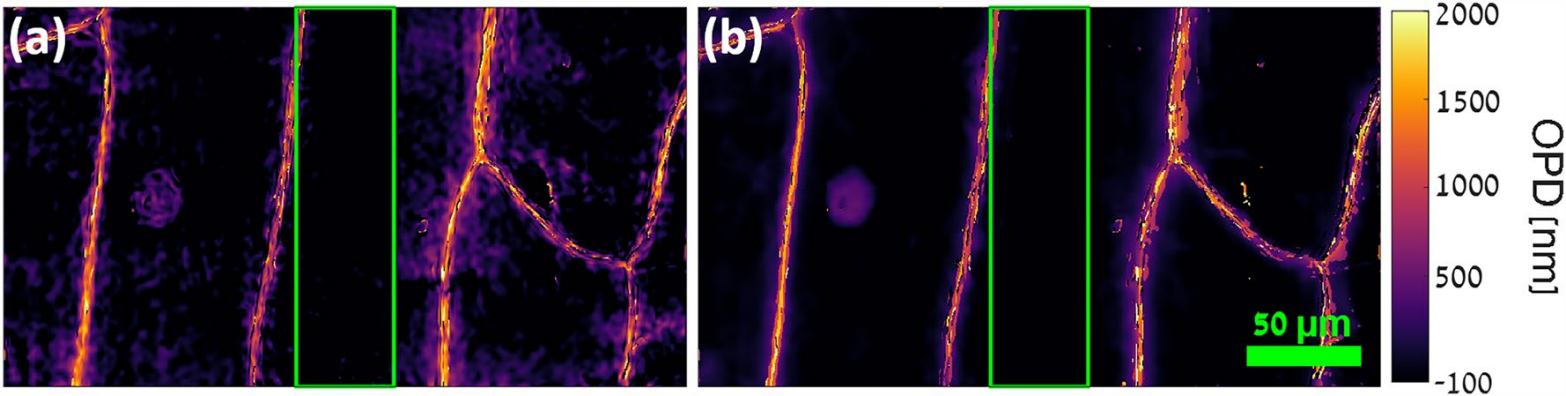
Unwrapped OPD maps of the combined FOVs of an onion epidermal tissue. (**a**) 6207 nm synthetic-wavelength OPD map, unwrapped by a standard unwrapper^38^. (**b**) Hierarchically-unwrapped OPD map. Scale and color bars apply to both phase maps. Green rectangles indicate region of overlap between FOVs.

In order to demonstrate the potential of the system for dynamic imaging and scanning of biological tissues, a monolayer of live onion (Allium cepa) epidermal tissue, with an estimated typical thickness of 140 μm^45^, was imaged. The tissue was immersed in phosphate buffer solution (PBS) and covered with a coverslip prior to imaging. The resulting wrapped phase maps are shown in Fig. 8, and corresponding unwrapped OPD maps are shown in Fig. 9, with a video of the sample during scanning shown in Visualization 1. Figure 9a shows the standard-unwrapped 6207 nm synthetic-wavelength OPD map, while Fig. 9b shows the hierarchically-unwrapped OPD map. The hierarchically-unwrapped OPD map displays less noise than the standard-unwrapped OPD map, and a nucleus is visible on the left. Interestingly, the OPD value within the cells is approximately ‒100 nm, indicating that the integral refractive index of these cells in the direction of the optical axis is approximately 0.0007 less than that of the PBS medium, assuming the previously mentioned typical onion-tissue thickness. Also of note are the high OPD values in the vicinity of the cell walls, appearing as bright channels in the figures, with a typical value of roughly 1600 nm and reaching up to 3000 nm. This is to be expected, as the cell walls are composed primarily of cellulose, which has a relatively high refractive index of 1.47^46^, and these cell walls are aligned nearly parallel to the optical axis and assumed to possess approximately the same thickness as the tissue, 140 μm. The values near the cell walls are also higher in the hierarchically-unwrapped OPD map compared to those obtained by the standard-unwrapped OPD map of 692 nm, which presents values of roughly 1000 nm in these regions, thus indicating that the synthetic-wavelength unwrapping enables the acquisition of more-accurate OPD values.

In Visualization 1, acquired at eight frames per second (fps), we can see that the hierarchically-unwrapped OPD maps of the combined FOVs can be acquired dynamically while scanning the sample.

## Conclusion

We have presented a new technique that uses holographic multiplexing of six parallel wavefront channels to accurately and dynamically unwrap optically thick samples across two different FOVs by using three illumination wavelengths to generate six synthetic wavelengths and perform hierarchical phase unwrapping. This technique enabled a roughly 89% increase in OPD value accuracy for the control sample of a microfluidic channel with a known height, as well as a 93% reduction in OPD noise relative to the OPD map of the longest synthetic wavelength prior to hierarchical phase unwrapping. The system is also capable of dynamically imaging highly scattering tissue samples and improving OPD value accuracy for such samples as well. The current iteration of the system suffers from relatively low frame rates for scattering samples due to the loss of intensity at the reference pinhole, necessitating the use of a filter in the sample arm to decrease the sample beam intensity accordingly. In addition, there exist points in the beams where intensity is too low for recording interference, due to minor damage to the diffractive beam splitter in the reference arm, shown in Supplementary Fig. 1, as well as low sample beam intensity in highly scattering regions. The issue of low intensity limiting frame rate can be resolved by replacing the diffractive beam splitter that produces 77 reference beams with a custom-made element that would produce only the 6 necessary beams, resulting in a greater than 12-fold increase in reference beam intensity, which would roughly match the sample beam intensity without the filter, allowing the system to reach up to 96 fps without further alteration. In addition, the transmission:reflection ratios of the 50:50 beam splitters may be adjusted to further optimize sample and reference beam intensities. Finally, highly scattering samples may be imaged in the Fourier conjugate plane rather than the image plane in order to achieve more-uniform sample illumination.

In the future, the dynamically acquired OPD maps may be used for faster scanning of thick histological samples to provide higher quality imaging data for deep learning classification and segmentation, as well as dynamic analysis of living samples that could previously not be accurately imaged due to their spatial sizes. The presented technique may also be used for generating dynamic height maps of reflective samples for optical metrology and silicon wafer inspection.

### Supplementary Information

Supplementary Video 1.

Supplementary Information.

## Data Availability

The datasets used and/or analyzed during the current study are available from the corresponding author on reasonable request.
