# Author Correction: Six-pack holography for dynamic profiling of thick and extended objects by simultaneous three-wavelength phase unwrapping with doubled field of view

**DOI:** 10.1038/s41598-024-52369-w

**Published:** 2024-01-19

**Authors:** Simcha K. Mirsky, Natan T. Shaked

**Affiliations:** Department of Biomedical Engineering, Tel Aviv University, 69978 Tel Aviv, Israel

Correction to: *Scientific Reports* 10.1038/s41598-023-45237-6, published online 07 November 2023

The Funding section in the original version of this Article was omitted. The Funding section now reads

“This article was funded by the Israel Science Foundation (ISF), and Tel Aviv University's Light-Matter Interactions (LMI) Center.”

The original Article has been corrected.

