# Supplementary material for: Six-pack holography for dynamic profiling of thick and extended objects by simultaneous three-wavelength phase unwrapping with doubled field of view
Source: Sci Rep. 2023 Nov 7;13:19293. doi: 10.1038/s41598-023-45237-6 (PMC10630357; doi:10.1038/s41598-023-45237-6)
Supplement: Supplementary file 2 — Supplementary Information. [file 41598_2023_45237_MOESM2_ESM.pdf]

Six-Pack Holography for Dynamic Profiling of Thick and Extended Objects by Simultaneous Three-Wavelength Phase Unwrapping with Doubled Field of View

Simcha K. Mirsky and Natan T. Shaked*

Tel Aviv University, Department of Biomedical Engineering, Tel Aviv, 69978, Israel
* nshaked@tauex.tau.ac.il

Supplementary information

Visualization 1. Dynamic video of unwrapped onion epidermal tissue OPD maps. Available online.

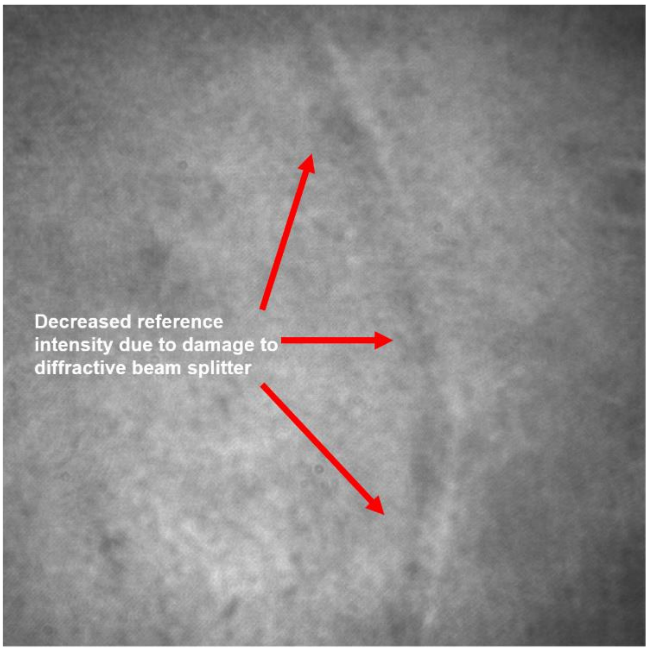

Supplementary Figure 1. Background reference hologram with fringes removed.
